# Multiobjective Sizing of an Autonomous Hybrid Microgrid Using a Multimodal Delayed PSO Algorithm: A Case Study of a Fishing Village

**DOI:** 10.1155/2020/8894094

**Published:** 2020-08-07

**Authors:** Raja Mouachi, Mohammed Ali Jallal, Fatima Gharnati, Mustapha Raoufi

**Affiliations:** ^1^Laboratory of Intelligent Energy Management and Information Systems, Faculty of Sciences Semlalia, Cadi Ayyad University, Marrakesh 40000, Morocco; ^2^I2SP Team, Physics Department, Faculty of Sciences Semlalia, Cadi Ayyad University, Marrakesh 40000, Morocco

## Abstract

Renewable energy (RE) systems play a key role in producing electricity worldwide. The integration of RE systems is carried out in a distributed aspect via an autonomous hybrid microgrid (A-HMG) system. The A-HMG concept provides a series of technological solutions that must be managed optimally. As a solution, this paper focuses on the application of a recent nature-inspired metaheuristic optimization algorithm named a multimodal delayed particle swarm optimization (MDPSO). The proposed algorithm is applied to an A-HMG to find the minimum levelized cost of energy (LCOE), the lowest loss of power supply probability (LPSP), and the maximum renewable factor (REF). Firstly, a smart energy management scheme (SEMS) is proposed to coordinate the power flow among the various system components that formed the A-HMG. Then, the MDPSO is integrated with the SEMS to perform the optimal sizing for the A-HMG of a fishing village that is located in the coastal city of Essaouira, Morocco. The proposed A-HMG comprises photovoltaic panels (PV), wind turbines (WTs), battery storage systems, and diesel generators (DGs). The results of the optimization in this location show that A-HMG system can be applied for this location with a high renewable factor that is equal to 90%. Moreover, the solution is very promising in terms of the LCOE and the LPSP indexes that are equal to 0.17$/kWh and 0.12%, respectively. Therefore, using renewable energy can be considered as a good alternative to enhance energy access in remote areas as the fishing village in the city of Essaouira, Morocco. Furthermore, a sensitivity analysis is applied to highlight the impact of varying each energy source in terms of the LCOE index.

## 1. Introduction

Electricity has become one of the most essential parts of modern life. Nowadays, the electricity sector is facing serious challenges, such as ensuring sufficient supply to keep up with the ever-increasing demand of electrical energy, reducing its costs, and limiting polluting emissions. For these reasons, the use of REs has become a global priority for most of the world's countries.

In this regard, the Moroccan government plans to decrease the use of fossil fuel energy by producing more than 40% of electrical energy using only clean and RE resources by 2020 [1]. Besides, they begin to take advantage of RE resources by exploiting the high potential of solar and wind resources through inaugurating several energy projects and implementing several research projects to extend the use of the RE resources. Currently, with these projects and considering the country's deployment of solar and wind energies, the national RE production will rise to 52% by 2030 [2]. Moreover, Moroccan climate is favorable for the installation of the medium and small scale A-HMG projects as a means to bring electricity production closer to consumption, limit investment in transmission, and reduce losses in distribution networks.

RE in the form of an A-HMG system offers a reliable and optimal cost-effective solution for centralized RE installations. A-HMG can operate in both off-grid and grid-connected configuration. However, A-HMG systems provide an opportunity to use the advantages of RE resources in combination with conventional ones [3].

In the recent literature, the primary goals are to formulate and implement energy production strategies with greater flexibility, higher efficiency, and lower LCOE. However, reaching these goals cannot be accessible due to the nonlinear behavior of RE production and storage systems, which are strongly dependent on weather changes. So, to overcome these problems, many optimization techniques are employed in several scientific contributions in the literature.

The optimization problem can result in a mono-objective or multiobjective problem, which can include the minimization of the LCOE, LPSP, and the pollutant emissions. Two frequently used optimization techniques are stochastic dynamic programming methods for optimizing the energy management problem with multidimensional objectives and metaheuristic techniques to solve the problem of optimization with multiconstraints, multidimensional, and highly nonlinear combinatorial problems.

Based on the literature review, we found several research contributions that are focused on the HMG systems optimization problems. The authors in [4] proposed a generalized formulation for intelligent energy management of a HMG in using multiobjective optimization to minimize the operation cost and the environmental impact, where they applied artificial neural network to predict RE generation and load demand. The problem formulation in this work includes optimal battery scheduling taking into account the uncertainty of the HMG exogenous variables and forecasted entities. However, the proposed approach, does not take into consideration the REF as an objective and the optimal sizing of battery storage is not taken into account.

In the research presented in [5], a technical and economic method is proposed to optimize a HMG based on mixed integer linear programming. In this paper, the cost function is solved via linear programming based on a general algebraic modelling system, where the optimization phase of the HMG system was performed via HOMER software. The authors have analyzed WT, PV, and biomass resource potential of a selected area in province Punjab, Kallar Kahar. The cost of energy is calculated for different peak load, energy demand profiles, and grid availability. However, the proposed formulations do not introduce any storage solution to solve the intermittence problem of RE and to minimize the emission of polluting gases by biomass resource.

Taha and Yasser [6] presented a robust algorithm based on a predictive control model for an isolated HMG in four different case studies to minimize the total operating cost, to minimize the pollutant CO_2_, and to minimize the dump energy. The results showed that the optimal battery daily number of cycles, minimum SOC, and the initial SOC of the battery storage depend the pool-price variation. Moreover, the number of scenarios required to achieve a good results makes the problem very large and time-consuming to solve.

A mixed method is proposed for microgrid energy management in [7]. This was achieved by using linear optimization methods to obtain an optimal energy storage system size. The grid-connected microgrid system consists of solar PV, two identical solid oxide fuel cells, and a battery bank operated parallel with the load. Moreover, the proposed methodology neglected many important constraints on the LPSP and REF indexes.

The authors in [8] analyzed an energy management system for a hybrid AC/DC microgrid in an isolated community that employs a PV system for desalination in order to minimize the daily operating costs, by using the mixed integer nonlinear programming. The aim of the proposed methodology is to manage all system assets to ensure stable operation of the hybrid AC/DC microgrid and secured clean water supply for the customers. This contribution has neglected the REF and the availability objectives.

Correa et al. [9] proposed an energy management system based on a virtual power plant. The studied microgrid has solar panels and storage systems and works in an interconnected manner. These elements are modeled using linear programming methods to minimize the operating costs. REs are incorporated into an energetic model and are mainly based on hydric resources. Moreover, the authors concluded that the battery sizing plays a crucial role in operational cost of the microgrid. However, this contribution has considered a single objective optimization problem, since the statement of a number of objective optimization problems affects the convergence speed and the stability of the adopted algorithms.

In the research presented in [10], an intelligent energy management system is proposed for HMG system in three stations in Iran. The objective for this study is to minimize the operational costs and environmental impacts by using a multiobjective particle swarm optimization algorithm. The LCOE and the LPSP are defined as objective functions. The result of optimization shows that HMG system can be applied for one of the three proposed locations with a high REF. However, the optimal configuration and the LCOE index are not a standard value, which depends on location.

The authors in [11] presented an energy management system for a PV/WT/DG stand-alone HMG, which is optimized using a particle swarm algorithm with Gaussian mutation. This paper minimizes both the capital and fuel costs of the system. However, the authors mainly concentrated on economic evaluation of hybrid energy systems but they do not examine other aspects of sustainability of these systems.

Katsigiannis et al. [12] used multiobjective optimization techniques to size an A-HMG while simultaneously minimizing LCOE and CO_2_ emissions. The obtained results of LCOE are not competitive.

The previous reviewed studies for A-HMG optimization have recorded the following gaps:

1. Few research contributions have considered more than two objective optimization problems considering the computational complexity that affects the convergence speed and the stability of the adopted algorithms.
2. Many studies have dealt with the techno-economic analysis of the A-HMG system, while the optimal configuration and the LCOE index are not a standard value, which depends on several variables such as location, weather, and the applied optimization technique.

Based on the results presented by the studies mentioned above, the prime aim of the present work is adopting an efficient variant of PSO algorithm referred to as a MDPSO, which is developed recently by Song et al. [2]. The MDPSO is applied to design an optimal A-HMG with a high REF and minimal values of the LCOE and the LPSP indexes.

The elements of innovation of the present work are the following:

1. A recent MDPSO metaheuristic algorithm was adopted for an A-HMG system optimization.
2. The stability and the convergence of the adopted MDPSO algorithm were evaluated to build A-HMG with a high REF and minimal values in terms of the LCOE and the LPSP indexes.
3. The application of MDPSO improves the convergence speed of the traditional PSO algorithm and decreases the probability of converging to local optimum. So, the entire search space will be explored efficiently.
4. The applied optimizer demonstrates a high efficiency to build an optimal A-HMG system for a fishing village that is located in the coastal city of Essaouira, Morocco.
5. The developed A-HMG system present minimal values in terms of the LCOE and the LPSP with a high renewable factor compared to different A-HMG models developed in the literature.

## 2. Autonomous Hybrid Microgrid System Description

**Figure 1:**
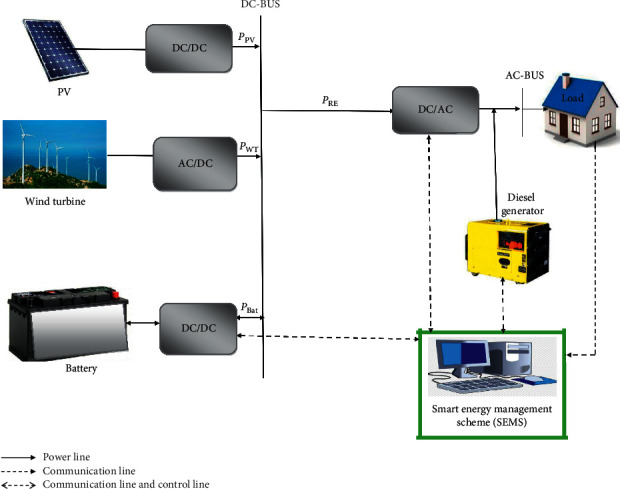
Schematic diagram of the A-HMG system.

The proposed A-HMG system is composed of wind turbines (WTs), photovoltaic (PV) panels, diesel generator (DGs), and battery storage bank as shown in Figure 1, which can vary greatly depending on specific parameters, such as the availability of renewable resources and desired services to provide [13]. These parameters have a high impact on decision-making, and, accordingly, on the LCOE and reliability of the system. In the following section, a detailed modelling of each component of A-HMG is introduced and discussed.

### 2.1. Photovoltaic Energy System Model

Morocco has a favorable climate conditions for implementing large solar energy projects. Various models for calculating the output power of the PV system have been proposed in the literature [2]. In this work, a simplified model that considers ambient temperature and solar irradiance is used, as shown in the following equation [10, 14]:

(1) $$\begin{array}{c} P_{\text{pv}}=\;G\times P_{r}\times \frac{\left[1+\;K\;\left(\left(\;T_{\text{amb}}+\left[\left(\text{TNOCT}-20\right)|/|800\right]G\right)\;-\;T_{\text{ref}}\;\right)\right]}{G_{\text{ref}}}, \end{array}$$

where

- *P*_pv_ and *P*_*r*_ are the output power of the PV and the rated power in watts (W) at the standard test condition (STC), respectively;
- *K* is the temperature coefficient, defined by (−3.7 × 10^−3^ (1/°C));
- *T*_ref_ denotes the cell temperature (°C) at STC;
- *T*_amb_ denotes the ambient temperature (°C) (*T*_amb_=25 °C );
- *G* is solar radiation (W/m^2^);
- *G*_ref_ is solar radiation at STC (*G*_ref__ = _1000 W/m^2^);
- TNOCT is the nominal operating cell temperature.

### 2.2. Wind Turbine Energy System Model

Wind energy offers enormous benefits for Morocco, where this energy is regarded as being abundant, climate-friendly, easy to operate, and cost-efficient. Additionally, the wind potential in Morocco is highly important due to the ideal geographical position of the country.

To determine the WT generator output power, the measured wind speed at reference height is first converted to corresponding WT hub height. The power-law equation is computed by the following correlation [10]:

(2) $$\begin{array}{c} \frac{V_{1}}{V_{2}}\;={\left(\;\frac{h}{h_{\text{ref}}}\right)}^{\alpha }, \end{array}$$

where

- *h* (m) is the WT hub height
- *h*_ref_ (m) is WT reference height (*h*)
- *V*_1_ (m/s) is the wind speed at WT hub height
- *V*_2_ (m/s) is the speed at the reference height (*h*_ref_)
- *α* is the power-law exponential (also known as Hellmann exponent, wind gradient, or power-law exponent)

The WT output power can be expressed as [4, 15, 16]

(3) $$\begin{array}{c} P_{\text{WT}}=\left\{\begin{array}{cc} 0, & \mathrm{if}\;V<V_{\text{cut}-\text{in}}\;,V>V_{\text{cut}-\text{out}},\\ \frac{P_{r}}{\left(V_{r}^{3}-V_{\text{cut}-\text{in}}^{3}\right)}\;\left[V^{3}-V_{\text{cut}-\text{in}}^{3}\right],\; & \mathrm{if}\;V_{\text{cut}-\text{in}}\leq V<V_{r},\\ P_{r}\;, & \mathrm{if}\;V_{r}\leq V<V_{\text{cut}-\text{out}}, \end{array}\right. \end{array}$$

where

- *P*_r_ (kW) is the rated power of the WT
- *V* (m/s) is the wind speed
- *V*_cut-out_ (m/s) is the cut-out speed of the WT
- *V*_r_ (m/s) is the nominal speed of the WT
- *V*_cut-in_(m/s) is the cut-in speed of the WT

**Figure 2:**
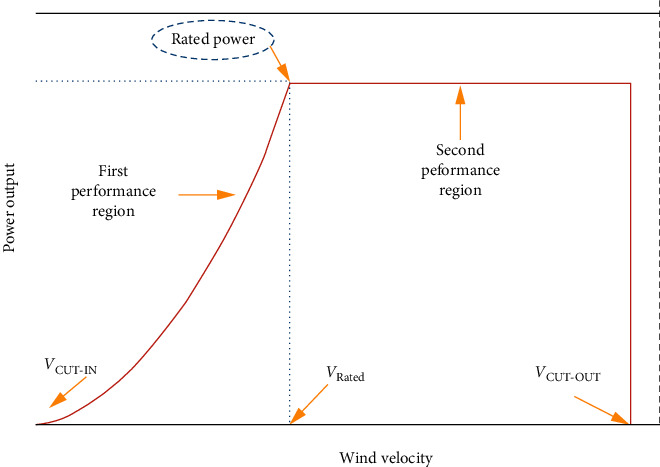
A typical WT power-wind speed characteristics [16].

Figure 2 portrays the power curve characteristics of a typical WT.

### 2.3. Battery Storage Bank Model

The battery capacity of the system is designed to meet the energy demand during periods of non-availability of renewable energy (RE) sources. In this regard, the battery capacity is designed in accordance with the desired autonomy day (AD) and with the energy demand *P*_load _ using the following equation:

(4) $$\begin{array}{c} C_{B}=\frac{P_{\text{load }}\times AD}{\eta_{\text{Bat}}\times \text{DOD}\times \eta_{\text{inv}}}, \end{array}$$

where

- DOD is the depth of discharge (80%)
- *η*_inv_ (95%) is the inverter efficiencies
- *η*_Bat_ (85%) is the battery efficiency

Moreover, the state of charge of a battery at any given time is SOC(*t*) and it is bounded by maximum SOC(SOC_max_) and minimum SOC(SOC_min_). The WT and PV systems could produce a surplus or deficit energy. A surplus/deficit energy represent the power absorbed/delivered by the battery storage system and it can be expressed as follows:

(5) $$\begin{array}{c} P_{\text{Bat}}\left(t\right)=\left(P_{\text{pv}}\left(t\right)+P_{\text{WT}}\left(t\right)\;\right)-\frac{P_{\text{load}}\left(t\right)}{\eta_{\text{inv}}}, \end{array}$$

where

- *P*_Bat_(*t*) < 0 is an indication that the power generation has deficit in energy demand
- *P*_Bat_(*t*) > 0 indicates a surplus in power generation

The battery storage bank charging occurs when SOC(*t*) < SOC_max_ and when (*P*_pv_(*t*)+*P*_WT_(*t*)>_ _*P*_load_(*t*)). Therefore, the SOC(*t*) can be expressed as follows [17]:

(6) $$\begin{array}{c} \text{SOC}\left(t\right)=\text{SOC}\left(t-1\right)\left(1-\gamma \right)+\left(P_{\text{Bat}}\left(t\right)\times \eta_{\text{Bat}}\right). \end{array}$$

Furthermore, when there is a deficiency in the power generation and SOC(*t*) > SOC_min_, the battery storage bank then discharges the energy stored into it to achieve the energy demand according to

(7) $$\begin{array}{c} \text{SOC}\left(t\right)=\text{SOC}\left(t-1\right)\left(1-\gamma \right)+\left(-P_{\text{Bat}}\left(t\right)\times \eta_{\text{Bat}}\right), \end{array}$$

where

- *γ* denotes the self-discharge rate of the battery storage bank

### 2.4. DC/AC Inverter Model

The inverter DC/AC converts the electrical energy from DC to AC with the desired frequency of the load. Its efficiency is defined by the following equation:

(8) $$\begin{array}{c} \eta_{\text{inv}}=\frac{P}{P+P_{0}+kP^{2}}, \end{array}$$

where *P*, *P*_0_, and *k* are determined by using the following equations [18–21]:

(9) $$\begin{array}{c} K=\;\frac{1}{\eta_{100}}-P_{0}-1,\\ P_{0}=1-99{\left(\frac{1}{\eta_{10}}-\frac{1}{\eta_{100}}-9\right)}^{2},\\ P=\frac{P_{\text{out}}}{P_{n}}, \end{array}$$

where *η*_10_ and *η*_100_ are the efficiency of the inverter at 10% and 100% of its nominal power, respectively.

### 2.5. Diesel Generator Model

Diesel generator (DG) works as a secondary source of energy in the case of battery depletion during peak energy demand.

The efficiency and the hourly fuel consumption of the diesel generator should be considered in designing a hybrid energy system and can be expressed by the following expression [22, 23]:

(10) $$\begin{array}{c} F\left(t\right)=aP_{\text{DG}}\left(t\right)+bP_{r}, \end{array}$$

where

- *P*_DG_ is the generated power (kW)
- *F*(*t*) is the fuel consumption (L/hour)
- *P*_*r*_ is the rated power (kW) of the DG
- *a* and *b* are constant parameters (L/kW), which represent the coefficients of fuel consumption, and can be approximated to 0.246 and 0.08415, respectively [24]

Similarly, the efficiency of the DG is calculated by the following equation [25]:

(11) $$\begin{array}{c} \eta_{\text{overall}}=\eta_{\text{brak}-\text{thermal}}\times \eta_{\text{generator}}, \end{array}$$

where

- *η*_overall_ represents the overall efficiency of the DG
- *η*_brak−thermal_ is the brake thermal efficiency of the DG

## 3. Site of Implementation and A-HMG System Descriptions

Moroccan lands are blessed with abundant renewable energy resources, predominantly those of solar and wind energies. In the present study, an A-HMG system was designed for a small fishing village located in a coastal region in the middle of Morocco, nearby the city of Essaouira at a latitude of 31.52°N and a longitude of 9.27°W.

**Figure 3:**
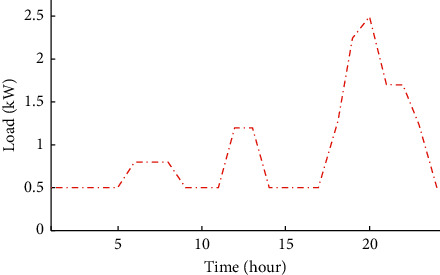
Load profile.

**Figure 4:**
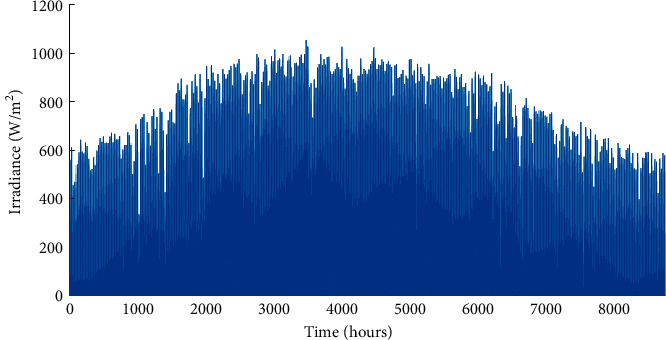
Hourly solar irradiance time series of the studied site.

**Figure 5:**
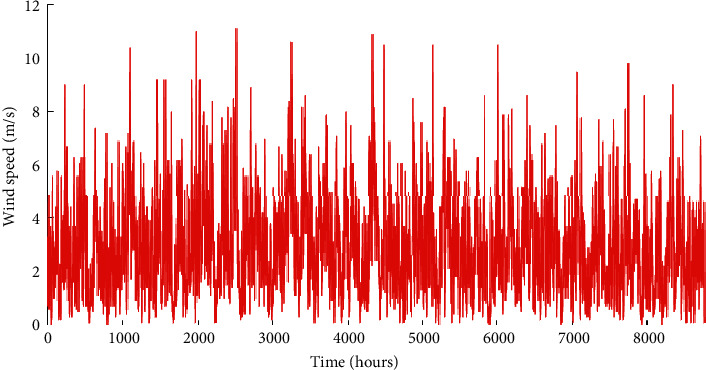
Hourly wind speed time series of the studied site.

**Figure 6:**
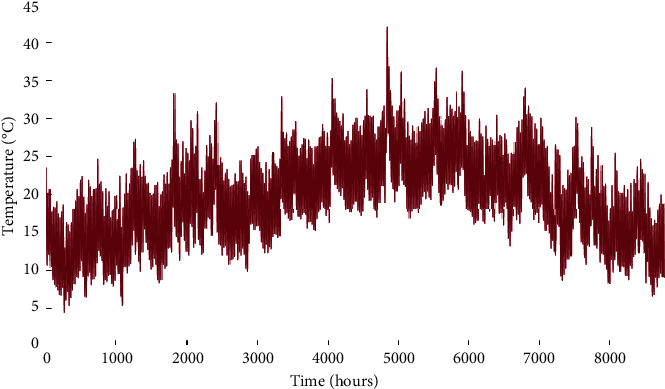
Hourly ambient temperature time series of the studied site.

This region is characterized by high irradiance levels and an average annual wind speed between 7 and 8 m/s [22, 26]. Its exposure to the Atlantic Ocean moderates the temperature amplitudes compared to inland regions. The economic and technical parameters of the A-HMG components are given in Table 1. The hourly load profile is given in Figure 3. Similarly, Figures 4–6 depict the hourly profiles of solar radiation, wind speed, and ambient temperature, respectively.

In general, the efficiency of the resources (PV, WT, DG, and battery) is reduced with time with a degradation factor. The energy generated by each resource is equal to the rated energy output multiplied by the degradation factor. However, the impact of degradation on system performance is taken into account in this work, where the PV and WT's degradation factor is considered as 0.5% per year and the DG's degradation factor is considered as 0.7% per year.

## 4. The Proposed Smart Energy Management Scheme

Having an efficient SEMS is one of the major criteria to be considered when designing the A-HMG system. The objective of the proposed SEMS system is to coordinate the power flow into the A-HMG system and to maximize the use of renewable energy systems. The proposed SEMS system is based on the following cases:

  Case 1: Sufficient energy is provided by PV and WT resources, which will supply the load demand. Then, the extra energy will be used to charge a battery storage bank if SOC(*t*) *<* SOC_max_.
  Case 2: The energy that is provided by PV and WT resources exceeds the load demand and the battery storage bank needs (SOC(*t*) *=* SOC_max_).Therefore, the extra energy will be consumed in a dump load.
  Case 3: The energy generated by PV and WT resources is not sufficient to supply the load demand and SOC(*t*) *>* SOC_min_. Therefore, the stored energy in the battery storage bank will be used to satisfy the load demand.
  Case 4: The energy generated by the PV and WT resources is not sufficient to fulfill load demand and the battery storage bank is also depleted (SOC(*t*) < SOC_min_). Therefore, the DG is switched on to supply the load demand and charge the battery storage bank. Moreover, when PV and WT resources start to generate power, the energy produced by DG will be stopped.

**Figure 7:**
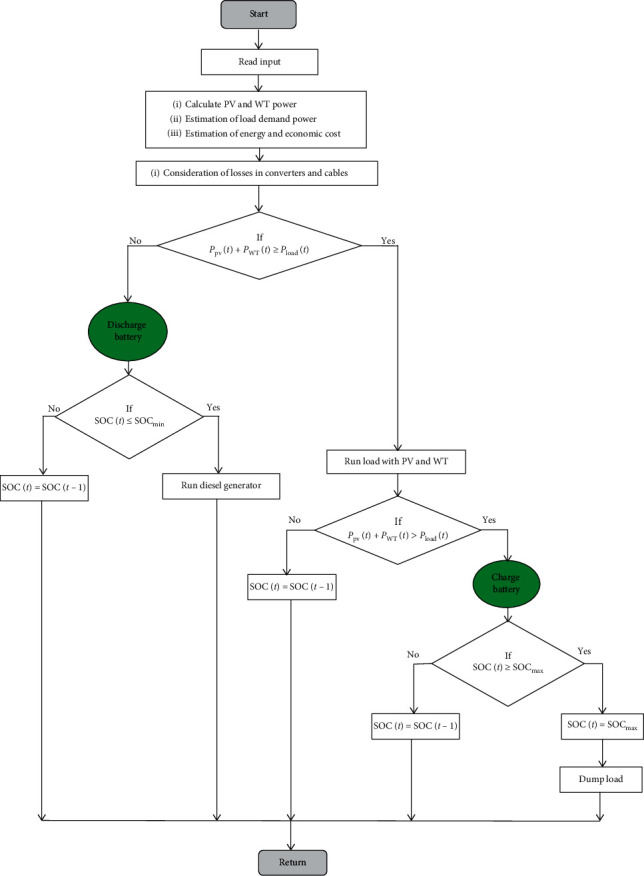
Main flowchart of the proposed SEMS.

The main flowchart for the proposed SEMS is shown in Figure 7.

## 5. Optimization Problem Formulation

The objective of this section is to provide an optimal configuration of A-HMG system to satisfy the typical load demand presented in Figure 3. The A-HMG system is optimized based on a multiobjective problem, which can be generally defined as follows:

(12) $$\begin{array}{c} \begin{array}{c} \text{Min }F\left(x\right)=\left[\begin{array}{c} f_{1}\left(x\right)\\ f_{2}\left(x\right)\\ \vdots \\ f_{k}\left(x\right) \end{array}\right]\\ \text{Subject to}\left\{\begin{array}{c} H\left(x\right)\leq 0,\\ G\left(x\right)=0, \end{array}\right. \end{array}, \end{array}$$

where

- *F*(*x*) is a vector representing the objective functions
- *f*_1_(*t*), *f*_2_(*t*),…, *f*_*k*_(*t*) are the individual values of the objective functions
- x is the vector of the design search space
- *H*(*x*) and *G*(*x*) denote the set of inequality and equality constraints, respectively

### 5.1. Objective Functions Descriptions

The A-HMG system is evaluated based on the LCOE, the LPSP, and REF. Thus, they are chosen as the objective functions, where the goal objective is to optimize the system so that it would guarantee reliable power supply at minimum cost and maximize the reliability of the A-HMG system in case of a blackout, avoiding power supply interruptions and their related costs.

#### 5.1.1. Levelized Cost of Energy

The LCOE is one of the most used indicators of economic profitability of A-HMG systems [23]. The LCOE includes the hybrid system hardware initial cost and the operation and maintenance costs. It is defined as the price per unit produced energy ($/kWh) and expressed with the following equation [27, 28]:

(13) $$\begin{array}{c} \text{LCOE}\left(\frac{\text{\$}}{\text{kWh}}\right)=\frac{\text{TPV}}{\sum_{t=1}^{8760}P_{\text{load}}\left(t\right)}\;\times \text{ CRF,} \end{array}$$

where

- TPV is the total present value of the whole system cost and includes the capital, replacement, operation, and maintenance costs
- *P*_load_(*t*) is the hourly amount of energy consumed
- CRF is the capital recovery factor; these can be expressed as follows:

(14) $$\begin{array}{c} \text{CRF}\left(\beta ,n\right)=\frac{\beta {\left(1+\beta \right)}^{n}}{{\left(1+\beta \right)}^{n}-1}, \end{array}$$

where

- *n* is the life span, typically equal to the life span of the PV panels [29]
- *β* is the discount rate considered in the economic appraisal of the proposed system

The discount rate is country-dependent and also subject to the investment stability of the energy source. This parameter may vary from 6% to 16% for renewable energy sources in Morocco [30]

#### 5.1.2. Reliability Analysis

The reliability of the A-HMG system is assessed based on the LPSP. The LPSP is a reliability index, which indicates the probability of the power supply failure to meet due to low renewable resources or technical failures to meet the energy demand. It can be described by the following equation [31, 32]:

(15) $$\begin{array}{c} \text{LPSP}=\frac{\sum_{t=1}^{8760}\left(P_{\text{load}}\left(t\right)-P_{\text{PV}}\left(t\right)-P_{\text{WT}}\left(t\right)+P_{\text{DG}}\left(t\right)+P_{{\text{Bat}}_{\text{min}}}\right)}{\sum_{t=1}^{8760}P_{\text{load}}\left(t\right)}, \end{array}$$

where

- *P*_WT_(*t*) is the amount of power generated by the wind turbine at time *t*
- *P*_pv_(*t*) is the amount of power generated by the PV system at time *t*
- *P*_DG_(*t*) is the amount of power generated by the diesel generator at time *t*
- *P*_load_(*t*) is the amount of power consumed at time *t*
- *P*_Bat_min__ is the minimum allowable storage capacity of the battery storage system

The LPSP is a reliability index, which indicates the probability of the power supply failure to meet the energy demand. The LPSP value must be less than 5% based on the literature.

In this work, the reliability evaluations are carried out in the worst conditions, when

(16) $$\begin{array}{c} P_{\text{load}}\left(t\right)>P_{\text{generate}}\left(t\right). \end{array}$$

### 5.2. Constraints

REF is defined for MDPSO programming as a boundary to determine the amount of energy coming from a DG as compared to the RE generators. The objective is to minimize the DG usage, reducing the CO_2_ emissions and reducing the cost of operation. Hence, the REF is computed as follows:

(17) $$\begin{array}{c} \text{REF}\left(\%\right)=\left(1-\frac{\sum_{t=1}^{8760}P_{\text{DG}}\left(t\right)}{\sum_{t=1}^{8760}\left(P_{\text{PV}}\left(t\right)+P_{\text{WT}}\left(t\right)\right)}\right)\times 100. \end{array}$$

The REF is bounded by 0 and 1, where REF = 0 means that the renewable energy is not used and REF = 1 means that the energy is produced only by renewable energy sources without using the DG.

In addition, the following constraints for the number of renewable energy generators (PV panels and WT), DG, and the autonomy days should also be satisfied:

(18) $$\begin{array}{c} 0\leq N_{\text{PV}}\leq N_{{\text{PV}}_{\max}},\\ 0\leq N_{\text{WT}}\leq N_{{\text{WT}}_{\max}},\\ 0\leq N_{\text{AD}}\leq N_{{\text{AD}}_{\max}},\\ 0\leq N_{\text{DG}}\leq N_{{\text{DG}}_{\max}}, \end{array}$$

where

- *N*_pv_ and *N*_WT_ are the number of PVs and WTs, respectively
- *N*_AD_ and *N*_DG_ are the number of DGs and autonomy days (AD), respectively
- *N*_PV_max__ and *N*_WT_max__ are the maximum number of PVs and WTs, respectively
- *N*_AD_max__ and *N*_DG_max__ are the maximum number of ADs and DGs, respectively

## 6. PSO Algorithm Evolution

### 6.1. PSO Algorithm

In the past few years, a large number of PSO-based approaches have been proposed for optimal sizing of the A-HMG system. PSO algorithm was developed by Eberhart and Kennedy in 1995 for hard optimization problems. The searching strategy of PSO algorithm is inspired by the social behaviors of birds flocking or fish schooling, where each particle of the swarm acts as a potential solution of certain optimization problem [3].

PSO algorithm is employed to intelligently select optimal parameters from N particles. The initialization matrix contains N particles dispersed in a search space of D-dimension.

At the *k*^th^ iteration in the searching process, the *i*^th^ particle stores its historical best position P_best_ as represented by (*p*_best_(*k*)_ _=_ _(*p*_*i*1_(*k*), *p*_*i*2_(*k*),…, *p*_*iD*_(*k*)), and its global best particle P_gbest_ as denoted by (*p*_gbest_(*k*) = (*p*_gbest1_(*k*), *p*_gbest2_(*k*),…, *p*_gbestD_(*k*)). The process of displacement of each particle is managed by three rules [33, 34]:

1. The particle tends to take the direction of its current velocity
2. The particle tends to move toward its best position
3. The particle tends to move to the best position reached by its neighbors

In fact, the position of the particle *i* will be updated to reach the global optimum based on the corresponding velocity vector. Furthermore, the velocity and the position vectors of the particle *i* at the iteration *k* will be updated as follows:

(19) $$\begin{array}{c} V_{i}\left(k+1\right)=wV_{i}\left(k\right)+c_{1}r_{1}\;\left(p_{\text{best}}\left(k\right)-x_{i}\left(k\right)\right)+\;c_{2}r_{2}\left(p_{\text{gbest}}\left(k\right)-x_{i}\left(k\right)\right),\\ x_{i}\left(k+1\right)=x_{i}\left(k\right)+V_{i}\left(k+1\right), \end{array}$$

where

- *k* denotes the number of the current iteration
- *w* denotes the inertia weight
- *c*_1_ and *c*_2_ are called the acceleration coefficients, respectively, cognitive and social parameters
- *r*_1_ and *r*_2_ are two uniformly distributed random numbers in the interval [0, 1] [2]

A variety of approaches has been proposed to improve the capability of the traditional PSO algorithm [35].

Shi et al. [36, 37] introduced PSO with linearly decreased inertia weight *w* on iteration generations; the inertia weight of the current iteration *w* is given as following equation:

(20) $$\begin{array}{c} w=\left(w_{\text{in}}-w_{\text{fi}}\right)\times \frac{{\text{it}}_{\max}-\text{it}}{{\text{it}}_{\max}}+w_{\text{fi}}, \end{array}$$

where

- *w*_in_ and *w*_fi_ denote, respectively, the initial value and the final value of the inertia weight
- it is the number of current iteration
- it_max_ denotes the number of maximum iteration

Overall, a larger inertia weight would make the PSO tend to the global exploration and, otherwise, a smaller one could achieve the local exploitation.

Hence, the initial and final values *w*_1_ and *w*_2_ are generally set as 0.9 and 0.4, respectively. Additionally, PSO with time-varying acceleration coefficients have been introduced in [2, 38] as calculated by the following equations:

(21) $$\begin{array}{c} c_{1}=\left(c_{1\text{i}}-c_{1\text{f}}\right)\times \frac{{\text{it}}_{\max}-\text{it}}{{\text{it}}_{\max}}+c_{1\text{f}}, \end{array}$$

(22) $$\begin{array}{c} c_{2}=\left(c_{2\text{i}}-c_{2\text{f}}\right)\times \frac{{\text{it}}_{\max}-\text{it}}{{\text{it}}_{\max}}+c_{2\text{f}}, \end{array}$$

where

- *c*_1i_ and *c*_2i_ are the initial values of the acceleration coefficients *c*_1_ and *c*_2_
- *c*_1f_ and *c*_2f_ are the final values of the acceleration coefficients

The improvement strategies of the traditional PSO mentioned above are principally concerning the parameter studies.

### 6.2. MDPSO Algorithm

In 2017, Song et al. developed a MDPSO algorithm for the global smooth path planning for mobile robots [2].

The principal novelty of the MDPSO algorithm as a new PSO algorithm is that it integrates two delayed terms in the traditional velocity. The updating terms are the local and global delayed best particles selected from the corresponding values in the previous iterations stochastically and are added into the velocity updating model according to the evaluated evolutionary state. The advantage of this method is to reduce the convergence speed of the traditional PSO algorithm and to decrease the probability of converging to local minimum [2].

The following equations calculate the updating equations for the velocity and the position of the novel MDPSO algorithm.

(23) $$\begin{array}{c} V_{i}\left(k+1\right)=wV_{i}\left(k\right)+c_{1}r_{1}\left(p_{\text{best}}\left(k\right)-x_{i}\left(k\right)\right)+c_{2}r_{2}\;\left(p_{\text{gbest}}\left(k\right)-x_{i}\left(k\right)\right)+S_{i}\left(k\right)c_{3}r_{3}\left(p_{\text{best}}\left(k-\tau_{i}\left(k\right)\right)-x_{i}\left(k\right)\right)+S_{g}\left(k\right)c_{4}r_{4}\;\left(p_{\text{gbest}}\left(k-\tau_{g}\left(k\right)\right)-x_{i}\left(k\right)\right),\\ x_{i}\left(k+1\right)=x_{i}\left(k\right)+V_{i}\left(k+1\right), \end{array}$$

where

- *w* is the inertia weight
- *c*_1_ and *c*_2_ are the coefficients for acceleration updated and *c*_3_ and *c*_4_ are equal to *c*_1_ and *c*_2_, where, *c*_1_=*c*_3_ and *c*_2_=*c*_4_, respectively
-  *r*_1_,*r*_2_,*r*_3_,*r*_4_ are the random uniformly distributed numbers in [0, 1]
- *τ*_*i*_(*k*) and *τ*_*g*_(*k*) are the random delays uniformly distributed in [0*, k*] for the local and the global delayed best particle, respectively
- *S*_*i*_(*k*) and *S*_*g*_(*k*) are the intensity factor of the newly added terms in the velocity updating model depending on the evolutionary factor

The newly added terms in the velocity updating model are closely related to the evolutionary factor (EF) defined in [39] to describe the swarm distribution properties. According to the EF in the searching process, the four states are denoted as follows: *ξ*(*k*) = 1 is the exploration state, *ξ*(*k*) = 2 is the exploitation state, *ξ*(*k*) = 3 is the convergence state, and *ξ*(*k*) = 4 the jumping out state, respectively. The mean distance between the particle *i* and the other particles in the swarm denoted as *d*_*i*_ could be calculated by

(24) $$\begin{array}{c} d_{i}=\frac{1}{S-1}\;\sum_{j=1,j\neq 1}^{S}\sqrt{\sum_{k=1}^{D}{\left(x_{ik}-x_{jk}\right)}^{2}}, \end{array}$$

where

- *D* is the particle dimension
- *S* is the swarm size

Therefore, the evolutionary factor (EF) is given by the following equation:

(25) $$\begin{array}{c} \text{EF}=\frac{{\text{di}}_{\text{gbest}}-{\text{di}}_{\min}}{{\text{di}}_{\max}-{\text{di}}_{\min}}, \end{array}$$

where

- di_gbest_ denotes the mean distance between the global best particle and the other particles in the swarm
- di_min_ denotes the minimum and the maximum of *d*_*i*_ in the swarm
- di_max_ denotes the maximum of *d*_*i*_ in the swarm

The evolutionary state is classified confer to the evolutionary factor by a series of fuzzy functions in [40]. Additionally, in [35, 41] equal division strategy is used for the evolutionary state classification for the state prediction depending on a Markov chain. The formulation of evolutionary used in this paper is expressed as follows [13]:

(26) $$\begin{array}{c} \xi \left(k\right)=\left\{\begin{array}{cc} 1, & 0\leq \text{EF}<0.25,\\ 2, & 0.25\leq \text{EF}<0.5,\\ 3, & 0.5\leq \text{EF}<0.75,\\ 4, & 0.75\leq \text{EF}\leq 1. \end{array}\right. \end{array}$$

Many strategies are used for the evolutionary states and the main idea of these strategies is to adjust the velocity updating model in an adaptive mode confer to the evaluated evolutionary state. In this work, a novel strategy with multimodal delayed information [2] is used to adaptively adjust the velocity updating model and expressed as follows:

- State 1: The particles in the swarm are expected to fly into the region around the global optimum as soon as possible. Therefore, only the normal terms in the velocity updating model remained and the delayed information is ignored (*m*_i_(*k*) = 0 and *m*_g_(*k*) = 0). This state is the convergence state, where *ξ*(*k*) = 1.
- State 2: The particles in the swarm are willing to exploit the region around the local best particles. So, the local delayed information is added into the velocity updating model; that is, only the local best particles in the previous iterations are randomly selected for the velocity updating with the intensity factor *m*_i_(*k*) = EF (*k*)). This state is the exploitation state, where *ξ*(*k*) = 2.
- State 3: In this state the global delayed information is added to explore the whole search space in a more thorough way, that is, the global best particles in the previous iterations are randomly selected for the velocity updating with the intensity factor *m*_*g*_(*k*) = EF(*k*) because it is important to search the optima as many as possible. This state is the exploration state where *ξ*(*k*) = 3.
- State 4: The local best particles are eager to jump out from the region around the local optima. Thus, it is necessary to provide more power for these particles to escape from this region, so that both of the local and global delayed information are used for this purpose with the intensity factor *m*_i_(*k*) = EF(*k*) and *m*_g_(*k*) = EF(*k*), respectively. This state is the state of jumping out, where *ξ*(*k*) = 4.

The above discussed states for multimodal delayed information can be summarized in Table 2 as follows.where

- *m*_i_(*k*) and *m*_g_(*k*) denote the intensity factor determined by the evolutionary state and evolutionary factor EF(*k*) in each iteration

**Figure 8:**
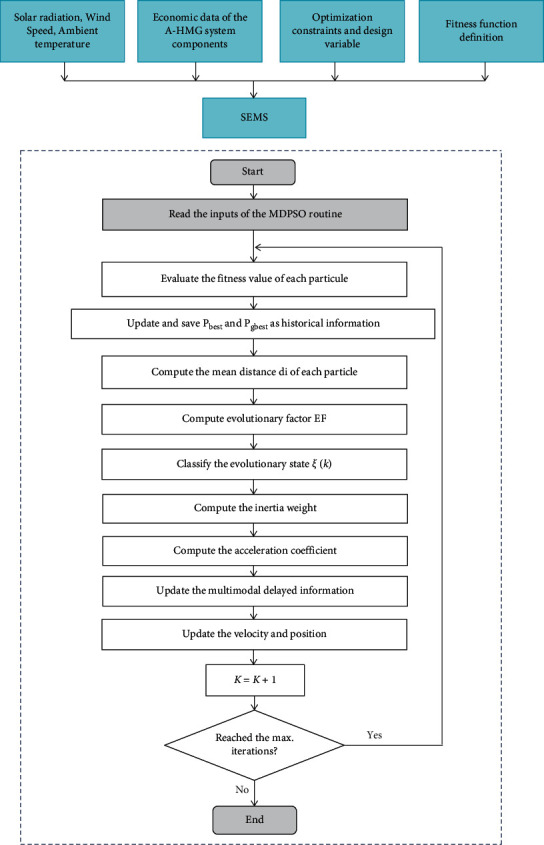
Flowchart of the proposed MDPSO algorithm.

The MDPSO algorithm flowchart is depicted in Figure 8.

## 7. Result Analysis and Discussion

### 7.1. MDPSO Parameters Configuration

The parameters configuration play a crucial role in manipulating the convergence speed of MDPSO algorithm. Therefore, there are many scientific researchers [2, 17, 42–44] to improve the PSO algorithm performances because these parameters should be selected carefully.

In Table 3, the parameters of the inertia weight and the acceleration coefficients are given by (20)–(22).

### 7.2. Results Analysis

In this section, the simulation results are presented. The SEMS for an A-HMG system is performed to fulfill the energy demand of a fishing village that is located in the coastal city of Essaouira, Morocco. The MDPSO method is applied to obtain the best configuration of A-HMG system and for sizing the components. The LCOE, LPSP, and REF are defined as objective functions. The studied system components are PV panels, WTs, battery storage bank, and DGs, as illustrated in Figure 1. The simulation conditions of the A-HMG optimization process over one year consider a community of 15 houses and the lifetime of the A-HMG has been chosen as 25 years.  Table 4 gives the best-found solution.

The obtained results in Table 4 show that we should install 15 units of PVs, 1 unit of WT, and 4 kW of DG. The minimum parameters come out to be 0.17 $/kWh for LCOE, 0.12% for LPSP, and 90% for REF. However, PV/WT/DG/battery as A-HMG system is the best configuration found for the fishing village site that is located in the city of Essaouira, Morocco.

The advantages of our system have more reliability and cost-effectiveness compared to those that use only one source of energy. These advantages are due to the combination of the proposed SEMS with the robust MDPSO algorithm.

**Figure 9:**
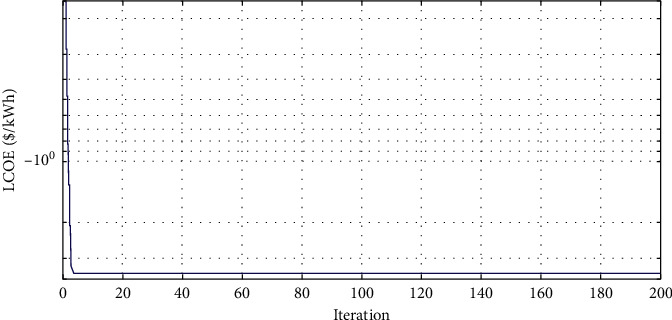
Convergence characteristic of MDPSO algorithm for the LCOE.

**Figure 10:**
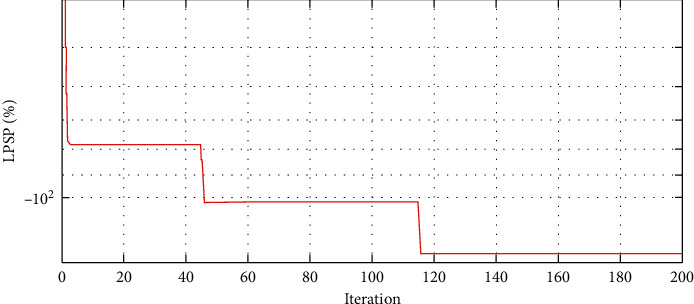
Convergence characteristic of MDPSO algorithm for the LPSP.

**Figure 11:**
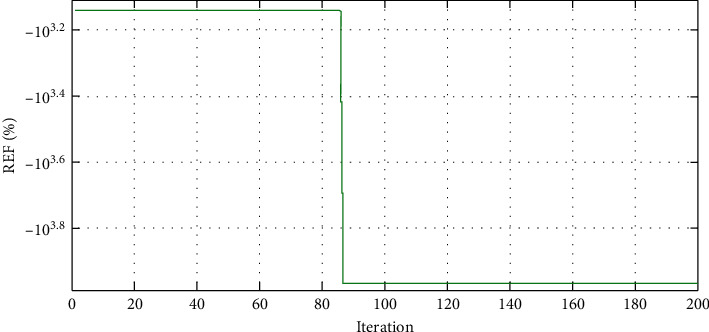
Convergence characteristic of MDPSO algorithm for the REF.

The convergence characteristics of MDPSO for the A-HMG are depicted in Figures 9–11 for the LCOE, the LPSP, and the REF, respectively. As seen in Figure 9, the value of the LCOE decreases during the iteration process and the same applies to the LPSP in Figure 10. Unlike the REF which increases as shown in Figure 11, we conclude that the MDPSO algorithm reduces the LCOE and the LPSP by moving toward the best system size. Moreover, any decline in the objective function leads to having more information about the optimal size.

These results will be confirmed through a sensitivity analysis to reveal the robustness of the optimization results.

### 7.3. Sensitivity Analysis

**Figure 12:**
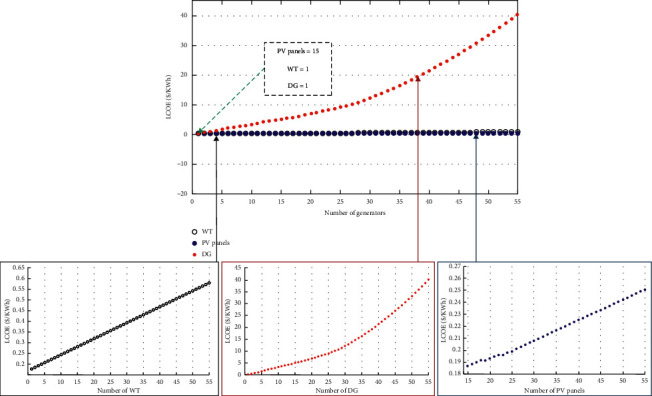
Sensitivity of LCOE to the variation of PV panels, WTs, and DGs numbers.

The sensitivity analysis is fundamental because it gives a better understanding of the effects of varying the input parameters of the A-HMG system. The analysis has been extended to qualify and quantify the effect of parametric variation in the LCOE for the A-HMG system, which is computed by the MDPSO algorithm. In addition, the LCOE values become more significant and more sensitive to lower numbers of PV panels, WTs, and DGs, which indicates that any change in the component size can change LCOE.  Figure 12**s**hows the impact of component resizing on the LCOE.

This shows that the LCOE savings of an A-HMG system can be enhanced with the integration of the additional number of green technologies but it increases with the integration of additional number of DGs. It is also important to note that the benefits of RE resources when compared with the brown technologies depend on the availability of the local RE resources, the capacity of the battery, and the level of RE technologies penetration.

**Figure 13:**
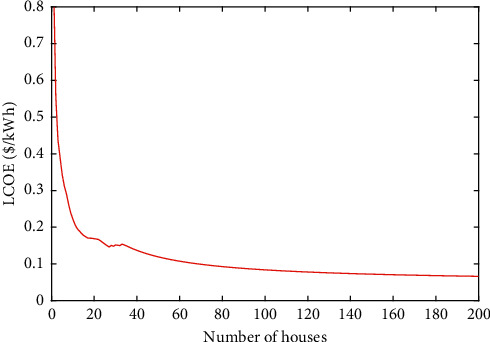
Sensitivity of LCOE to the variation of the houses number.

In Figure 13, the effect of LCOE was analyzed due to the variation of the number of houses in the village. It can be observed from the graph that the number of houses in the village has a nonlinear effect on LCOE.

### 7.4. Comparison of LCOE Results with Similar HMG Analyses in the Literature

Table 5 shows the comparison of LCOE with those available in literature. The achieved value of the LCOE index in this work is very interesting compared to those recently reported in the literature, regarding the A-HMG system optimization. The LCOE can significantly differ from country to another. However, this comparison is valuable as it can give an insight regarding the economic feasibility of A-HMG system in the city of Essaouira, Morocco, and for other sites across the coastal regions of Morocco.

The developed A-HMG system presents minimal values in terms of the LCOE and the LPSP with a high REF compared to different A-HMG models developed in the literature [3, 39, 40, 45–48]. So, few studies have considered more than two objective optimization problems considering the computational complexity that affects the convergence speed and the stability of the adopted algorithms. However, many research contributions have dealt with the technoeconomic analysis of the A-HMG system, while the optimal configuration and the LCOE index are not a standard value, which depends on several variables such as location, weather, and the applied optimization technique, the case of some studies in literature [39].

Moreover, the present work uses a MDPSO algorithm that improves the traditional PSO algorithm's research strategy and decreases the probability of converging to local optimum. Thus, the entire search space will be explored efficiently, contrary to other works in literature that used the conventional PSO algorithm for the optimization problem [48]. The MDPSO is a specific algorithm that gives to the user the ability to modify and add more sophisticated mathematical models for various RE technologies unlike the commercial software tools that restrict the user [46]. Finally, this paper does not used many numbers of scenarios that are required to achieve a good results, where the high number of scenarios makes the problem very large and time-consuming to solve [47].

## 8. Conclusions

In this paper, a smart energy management scheme (SEMS) with a multimodal delayed particle swarm optimization (MDPSO) is developed for performing the optimal sizing for the A-HMG system of a fishing village in the city of Essaouira, Morocco. The developed A-HMG system model contains PV panels, WTs, DGs, and battery storage systems. The levelized cost of energy (LCOE), the lowest loss of power supply probability (LPSP), and the maximum renewable factor (REF) are defined as objective functions. The results of the optimization in this location show that A-HMG system can be applied for this location with a high renewable factor. Moreover, the solution is very promising in terms of achieving values of the LCOE (0.17 $/kWh), LPSP (0.12%), and the REF (90%). Therefore, using renewable energy can be considered as a good alternative to enhancing energy access in remote areas as the fishing village in the city of Essaouira, Morocco. The obtained results demonstrate a high efficiency of the successful application of the MDPSO for the proposed SEMS compared to several research contributions in the literature. The utilization of the proposed method can help to overcome some of the technical problems that still limit the distribution of A-HMG system projects in Morocco. Finally, sensitivity analysis is also carried out in order to verify the performance of the results.

## Figures and Tables

**Table 1:** Technoeconomic data of the considered power systems.

System parameters	Values
*A. Pv unit*	
Life time (years)	25
Initial cost [$/kW]	2150
Rated power (W)	275
DC/DC converter efficiency (%)	95
PV regulator cost [$]	1500

*B. wind turbine*	
Life time (years)	25
Initial cost [$/kW]	2000
Blades diameter (m)	6.4
Cut-in speed (m/s)	2.5
Cut-out speed (m/s)	40
Swept area (m^2^)	128.6
AC/DC converter efficiency (%)	95
Rated power (kW)	5
Wind turbine regulator cost [$]	1000

*C. Diesel generator*	
Life time (hours)	24,000
Initial cost [$/kW]	1000
Rated power (kW)	4

*D. battery*	
Life time (years)	12
Initial cost [$/kWh]	280
Rated power (kWh)	40
Efficiency (%)	85

*E. inverter*	
Life time (years)	25
Initial cost [$]	2500
Efficiency (%)	92

*F. economic parameters*	
Project life time (years)	25
Discount rate (%)	6
O&M + running cost (%)	20

**Table 2:** States for multimodal delayed information.

State	Mode	*mi*(*k*)	*m*_g_(*k*)
Convergence state	*ξ*(*k*) = 1	0	0
Exploitation state	*ξ*(*k*) = 2	EF(*k*)	0
Exploration state	*ξ*(*k*) = 3	0	EF(*k*)
Jumping out state	*ξ*(*k*) = 4	EF(*k*)	EF(*k*)

**Table 3:** Inertia weight and acceleration coefficients used in the present work.

Parameters	Reference	Value
Acceleration coefficients	[44]	*c*_1i_=2.5
		*c*_1f_ = 0.5
		*c*_2i_ = 0.5
		*c*_2f_ = 2.5

Inertia weight	[17]	*w*_in_=0.9
		*w*_fi_=0.4

**Table 4:** Optimal results obtained.

Location	Parameters	PV/WT/DG/battery
Essaouira (Morocco)	Number of iterations	200
	Number of particles	100
	Number PV panels	15
	Days of autonomy	5
	Number of WTs	1
	DG capacity (kW)	4
	LPSP (%)	0.12
	LCOE ($/kWh)	0.17
	REF (%)	90

**Table 5:** Comparison of LCOE with other studies available in the literature.

Reference	HMG components	Location	LCOE ($/kWh)	Optimization technique
[45]-2016	PV/WT/DG	Singapore (Singapore)	0.29	Distributed mutated particle swarm optimization

[39]-2016	PV/WT/FC	Nikouyeh (Iran)	0.56	Computer program
		Ghadamgah (Iran)	0.799	
		Moaleman (Iran)	0.54	
		Marvdasht (Iran)	0.62	

[3]-2018	PV/WT/DG/battery	Riyadh (Saudi Arabia)	0.33	Cuckoo Search algorithms

[46]-2018	PV/WT/DG/LB	Chhattisgarh (India)	0.27	HOMER software

[40]-2018	PV/WT/battery	Gökçeada (Turkey)	1.90	HOMER software

[47]-2019	PV/WT/LB/BDG/FC/H_2_	Dickinson (US)	0.37	Genetic Algorithm
		Lubbock (US)	0.41	
		Tucson (US)	0.43	

[48]-2020	PV/WT/DG/battery	Baghdad (Iraq)	0.2330	PSO algorithms
		Rabat (Morocco)	0.3214	

This study	PV/WT/DG/battery	Essaouira (Morocco)	0.17	MDPSO

LB : lead acid battery, BDG : biodiesel generators, FC : fuel cell, and H_2_: hydrogen.

## Data Availability

The typical years of solar radiation, wind speed, and ambient temperature time series are obtained using Meteonorm software.
